# Hypoxia-cultured human adipose-derived mesenchymal stem cells are non-oncogenic and have enhanced viability, motility, and tropism to brain cancer

**DOI:** 10.1038/cddis.2014.521

**Published:** 2014-12-11

**Authors:** Y Feng, M Zhu, S Dangelmajer, Y M Lee, O Wijesekera, C X Castellanos, A Denduluri, K L Chaichana, Q Li, H Zhang, A Levchenko, H Guerrero-Cazares, A Quiñones-Hinojosa

**Affiliations:** 1 Department of Neurosurgery and Oncology, Johns Hopkins University School of Medicine, Baltimore, MD, USA; 2 Department of Pharmacology, School of Medical Science and Laboratory Medicine, Jiangsu University, Zhengjiang, Jiangshu, People's Republic of China; 3 Department of Neurosurgery, Tongji Hospital, Tongji Medical College, Huazhong University of Science and Technology, Wuhan, Hubei, People's Republic of China; 4 Department of Molecular Microbiology and Immunology, Bloomberg School of Public Health, Johns Hopkins University, Baltimore, MD, USA; 5 Department of Engineering and Applied Science, Yale University, New Haven, CT, USA

## Abstract

Adult human adipose-derived mesenchymal stem cells (hAMSCs) are multipotent cells, which are abundant, easily collected, and bypass the ethical concerns that plague embryonic stem cells. Their utility and accessibility have led to the rapid development of clinical investigations to explore their autologous and allogeneic cellular-based regenerative potential, tissue preservation capabilities, anti-inflammatory properties, and anticancer properties, among others. hAMSCs are typically cultured under ambient conditions with 21% oxygen. However, physiologically, hAMSCs exist in an environment of much lower oxygen tension. Furthermore, hAMSCs cultured in standard conditions have shown limited proliferative and migratory capabilities, as well as limited viability. This study investigated the effects hypoxic culture conditions have on primary intraoperatively derived hAMSCs. hAMSCs cultured under hypoxia (hAMSCs-H) remained multipotent, capable of differentiation into osteogenic, chondrogenic, and adipogenic lineages. In addition, hAMSCs-H grew faster and exhibited less cell death. Furthermore, hAMSCs-H had greater motility than normoxia-cultured hAMSCs and exhibited greater homing ability to glioblastoma (GBM) derived from brain tumor-initiating cells from our patients *in vitro* and *in vivo.* Importantly, hAMSCs-H did not transform into tumor-associated fibroblasts *in vitro* and were not tumorigenic *in vivo*. Rather, hAMSCs-H promoted the differentiation of brain cancer cells *in vitro* and *in vivo*. These findings suggest an alternative culturing technique that can enhance the function of hAMSCs, which may be necessary for their use in the treatment of various pathologies including stroke, myocardial infarction, amyotrophic lateral sclerosis, and GBM.

Mesenchymal stem cells (MSCs) are multipotent cells, isolated from the bone marrow, adipose tissue, and muscle, among others. They are clonally expansive, with the capacity to differentiate into osteocytes, adipocytes, and chondrocytes.^1, 2^ MSCs are widely studied for their regenerative potential, tissue preservation capabilities, anti-inflammatory properties, and anticancer therapeutic potential.^3, 4, 5^ MSCs can serve as vehicles for delivering effective targeted therapy to primary brain cancer and metastatic cancer.^6, 7, 8^

Notwithstanding aggressive treatment of primary brain cancer (glioblastoma (GBM)) with surgical resection, chemotherapy, and radiotherapy, the median survival following diagnosis is 14.6 months.^9, 10, 11, 12, 13, 14, 15^ GBM-targeted therapy using neural stem cells and MSCs as vehicles for therapeutic agents is a promising strategy.^16^ MSCs seem to be the ideal stem cells, as they are autologous, easily collected, and easily re-implanted.^17, 18^ The most commonly used MSCs are bone marrow-derived MSCs (BM-MSCs) and human adipose-derived MSCs (hAMSCs). Compared with BM-MSCs, hAMSCs are easier to obtain.^19, 20^

Despite the potential utility of hAMSCs, their use is hampered by their low concentration within tissues.^21, 22^ Thus, *in vitro* expansion of hAMSCs is necessary. Compared with BM-MSCs, hAMSCs are more genetically and morphologically stable in long-term culture.^19, 20^ Nevertheless, current culturing conditions for both BM-MSCs and hAMSCs show a progressive decrease in viability and proliferative ability, and an increase in senescence ratio for these stem cells with time.^23, 24, 25, 26, 27, 28, 29^ Typically, hAMSCs are cultured under ambient conditions with 21% oxygen *in vitro*.^30^ However, physiologically, hAMSCs exist at much lower oxygen tensions, between 1 and 14%.^31, 32^ As a result of the limitations of culturing under normoxia, we investigated the effect of hypoxia on intraoperatively obtained hAMSCs by assessing proliferation, survival, differentiation, tumor formation, tumor tropism, and migration *in vitro* and *in vivo* in a rodent model with a human brain cancer. hAMSCs have been reported to transform into tumor-associated fibroblasts (TAFs), which can potentially support tumor growth and promote malignant phenotypes.^33, 34^ Yet, no studies have reported on the changes that may occur in hypoxia-cultured hAMSCs after they are exposed to brain cancer, both *in vitro* and *in vivo*. An understanding of the effects of hypoxia on hAMSCs^35^ is critical for its potential therapeutic applications including in the treatment of brain tumors, stroke, neuro-degenerative diseases such as multiple sclerosis, and dementia (Figure 1a).

## Results

### hAMSCs cultured in hypoxia remain multi-potent

**Figure 1:**
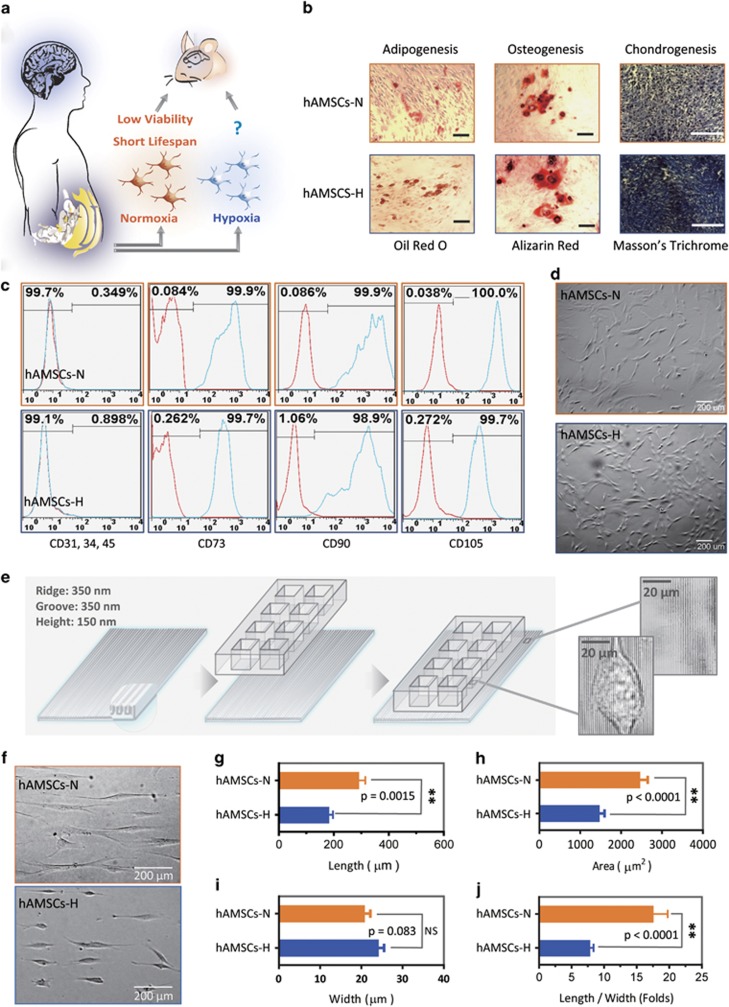
Primary human adipose-derived cells cultured in hypoxia (hAMSCs-H) and normoxia (hAMSCs-N) are both MSCs but normoxia-cultured cells show increased signs of senescence, such as increased area and elongated morphology, compared with hypoxia-cultured cells. (**a**) hAMSCs were isolated from human fat tissue and cultured in hypoxic (1.5% oxygen) or normoxic (21% oxygen) conditions *in vitro*. The viability, mobility, tumor tropism, safety, and tumorigenic potential were subsequently compared *in vitro* and *in vivo*. (**b**) Differentiation assay. hAMSCs were cultured in control media and differentiation media for 3 weeks, 10 days after the second passage. Three different stains were performed to assess differentiation capabilities (scale bar, 100 *μ*m). (**c**) Flow cytometric analysis was performed to confirm the absence of CD31-, CD34-, and CD45-positive cells in both cell cultures. In addition, primary hAMSC cultures expressed high levels of CD73, CD90, and CD105, both in hypoxic and normoxic culture conditions at day 10 after passage 2. (**d**) Representative images of cell morphologies of hAMSCs on 2D surface (scale bar, 200 *μ*m). (**e**) Schematic of 3D-nanopatterned surface used to assess morphology and motility. (**f**) Images of cell morphologies of hAMSCs on 3D-nanopatterned surface (scale bar, 200 *μ*m). (**g**–**j**) The length, width, area, and length-to-width ratio were measured and compared after cell aligned on the nanopattern surface. Error bars represent S.E.M. *P<0.05, **P<0.01, N.S., not significant

Multi-potency is critical to the therapeutic potential of hAMSCs. Primary hAMSCs cultured under hypoxia (hAMSCs-H) and normoxia (hAMSCs-N) remained multipotent, retaining the ability to differentiate into adipogenic, osteogenic, and chondrogenic lineages *in vitro* (Figure 1b). hAMSCs-H remained negative for CD31, CD34, and CD45 (≤2% positive), and expressed high levels of CD73, CD90, and CD105 (≥95% positive) (Figure 1c) via flow cytometric analysis, consistent with previous reports.^2^ We did observe slightly higher CD90 expression levels in hAMSCs-N compared with hAMSCs-N (99.9% *versus* 98.9%), which may have been due to the larger size of hAMSCs-N with the similar surface density of CD90 expression. However, hAMSCs-H and hAMSCs-N exhibited different phenotypes (Figure 1d). To quantify the difference in phenotype, both hAMSCs-N and hAMSCs-H were seeded onto a three-dimensional (3D)-nanopatterned surface to evaluate cell alignment and morphology (Figure 1e). hAMSCs-N displayed signs of senescence, including larger cell body areas and longer cell processes than hAMSCs-H (Figures 1f–j). Similar findings were seen in commercial hAMSCs-H (Supplementary Figures 2A–H).

### hAMSCs cultured in hypoxia grow faster, have higher viability, and passage for more generations than in normoxic conditions

**Figure 2:**
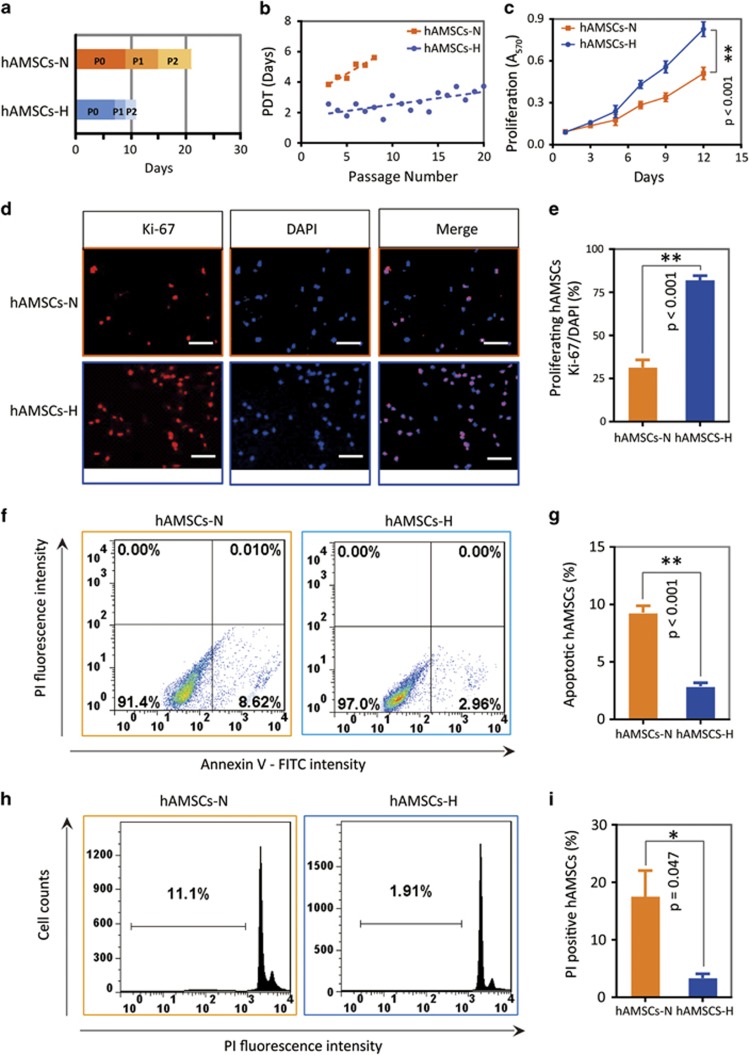
Primary hAMSCs cultured in hypoxia grow faster, have higher viability, and can be passaged for more generations *in vitro*. (**a**) hAMSCs-H had shorter P1 and P2 passage times. The passage time of P0 was calculated once cells reached 80% confluence, after 2 g of fat tissue was digested and seeded at a density of 0.6 × 10^4^/cm^2^ in hypoxic or normoxic conditions separately. The passage time of P1 and P2 was determined when 80% confluence was achieved. (**b**) Passage doubling time assay. hAMSCs-H had a constant PDT (<4 days) for up to 20 passages. PDT=*τ* Ln (2)/Ln (*N*_*τ*_/*N*_0_), where *τ*=time from plating to counting the cells, *N*_*τ*_=number of cells when counted and *N*_0_=initial number of cells. (**c**) MTT assay was used to determine the effects of hypoxia on the proliferative capacity of hAMSCs. (**d** and **e**) Ki-67 immunostaining was performed to quantify the number of proliferating cells. Representative images (scale bar, 100 *μ*m) and proliferating percentage of hAMSCs are shown. (**f** and **g**) Flow cytometric apoptosis analysis for Annexin V–FITC-positive and PI-negative cells. Representative histograms and quantification are shown. (**h** and **i**) PI staining was used to test the cell cycle and necrosis percentage of the cells. Representative histograms and quantification are shown. Error bars represent S.E.M. *P<0.05, **P<0.01, N.S., not significant

hAMSCs are known to have decreased proliferative capacity in current normoxic cell culture conditions, which will be a limitation for developing cellular therapies. The passage times of P0, P1, and P2 from nine primary hAMSC cultures in normoxia and hypoxia were compared. The passage times at P1 and P2 in hypoxic conditions were shorter than in normoxic conditions (*P*<0.05) (Figure 2a). In addition, the population doubling time (PDT) was also assessed. The PDT of hAMSCs-H remained more constant than hAMSCs-N over time (hAMSCs-H, *Y*=0.08 × *X*+2, *R*^2^=0.5; hAMSCs-N, *Y*=0.3 × *X*+3, *R*^2^=0.9) (Figure 2b). Moreover, hAMSCs-H could be passaged for more than 20 generations before the PDT reached 4 days as compared with only 4 generations for hAMSCs-N (Figure 2b). Similar results regarding passage times (P1 and P2) were found in other primary and commercial cell cultures (Supplementary Figure 3).

Next, we examined hAMSC viability and proliferation. hAMSCs-H grew faster than hAMSCs-N over 12 days in culture according to MTT assay (*P*<0.001; Figure 2c). Moreover, hAMSCs-H had a higher rate of proliferation based on Ki-67 calculations (Figures 2d and e). Furthermore, cell apoptosis was assessed using flow cytometry-based Annexin V–propidium iodide (PI). The proportion of apoptotic cells was smaller in hAMSCs-H than hAMSCs-N (*P*<0.001; Figures 2f and g). In addition, there were fewer cells in the pre-G1 phase of the cell cycle in hAMSCs-H compared with hAMSCs-N (Figures 2h and i; *P*=0.047). Similar results for passage time, PDT, MTT proliferation, Ki-67 percentage, apoptosis, and pre-G1 phase percentage were seen in commercial hAMSCs (Supplementary Figure 4). These results demonstrate that cell viability and proliferative ability were significantly higher in hypoxic conditions compared with normoxic conditions.

### Hypoxia-cultured hAMSCs retain their proliferation advantage over normoxia-cultured hAMSCs and their stem cell characteristics when exposed to GBM

**Figure 3:**
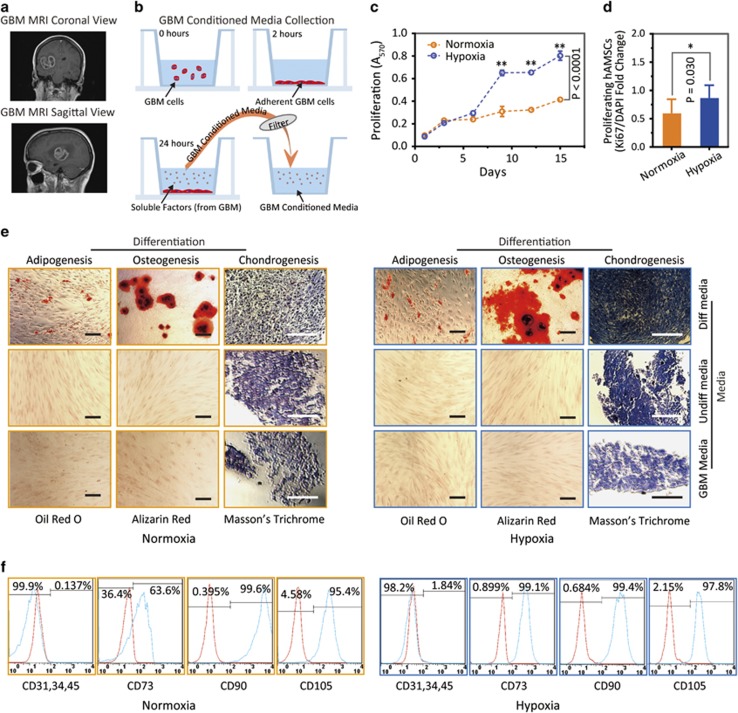
Hypoxia-cultured primary human adipose-derived mesenchymal stem cells (hAMSCs-H) retain a greater proliferation capacity compared with normoxia-cultured primary hAMSCs (hAMSCs-N) when exposed to GBM media. hAMSCs-H maintain stem cell characteristics when exposed to GBM media. (**a**) Representative MRI of GBM from a patient. (**b**) Schema showing the collection of GBM CM and culture of hAMSCs in filtered GBM CM for proliferation and migration assays. (**c**) MTT assay was used to determine the effects of hypoxic conditions on the proliferative capacity of primary hAMSCs in GBM CM. In GBM CM, hAMSCs-H showed greater proliferation at day 10 and 15 compared with hAMSCs-N. (**d**) Ki-67 immunostaining was performed to quantify the number of proliferating cells in GBM CM. Proliferative capacities of hAMSCs-H and hAMSCs-N are shown in GBM CM (normalized to hAMSC-N proliferative capacity in control media). In GBM CM, hAMSCs-H had a greater proportion of proliferating cells than hAMSCs-N. (**e)** Differentiation assay. hAMSCs were cultured in control media, differentiation media, and GBM CM for 3 weeks, 10 days after the second passage. Three stainings were performed to assess the differentiation capabilities (scale bar, 100 *μ*m). Both hAMSCs-N and hAMSCs-H maintained tri-lineage differentiation capability in GBM CM. (**f**) Flow cytometric analysis for CD31, CD34, CD45, CD73, CD90, and CD105 in hAMSC-N and hAMSC-H cultures after exposure to GBM CM for 20 days. hAMSCs-H maintained MSC phenotypic markers in GBM CM (negative for CD31, CD34, and CD45, and positive for CD73, CD90, and CD105). Error bars represent S.E.M. Error bars represent S.E.M. *P<0.05, **P<0.01, N.S., not significant

We examined hAMSC proliferative capacity and multipotency after exposure to GBM conditioned media (CM). This is essential for their therapeutic potential in the context of GBM treatment (Figure 3a). GBM CM from GBM276 was first collected (Figure 3b). hAMSCs-H grew faster than hAMSCs-N at day 10 (*P*<0.001) and day 15 (*P*<0.001) by MTT assay (Figure 3c and Supplementary Figure 5A). There was greater proliferation in hAMSCs-H *versus* hAMSCs-N after exposure to GBM CM, according to Ki-67 expression (*P*=0.03; Figure 3d and Supplementary Figure 5B).

Next, we examined hAMSC stem cell characteristics after exposure to GBM CM. Both hAMSCs-H and hAMSCs-N remained multipotent when cultured in GBM CM as evidenced by their ability to differentiate into adipogenic, osteogenic, and chondrogenic lineages *in vitro* (Figure 3e). hAMSCs-H cultured in GBM CM remained negative for CD31, CD34, and CD45, while expressing high levels of CD73, CD90, and CD105, consistent with International Society for Cellular Therapy (ISCT) criteria for MSCs.^2^ However, although hAMSCs-N also remained negative for CD31, CD34, and CD45 while expressing high levels of CD90 and CD105, they did not express high levels of CD73, and thus did not meet strict ISCT flow cytometric criteria for MSCs (63.6%, high level defined as ≥AMS expression) (Figure 3f).

### hAMSCs promote GBM differentiation *in vitro* and *in vivo*, and do not increase the proliferation or migration of GBM cells

It is critical that hAMSCs do not enhance brain tumor-initiating cell activity, GBM cell proliferation, or GBM cell migration to guarantee their safety as a potential therapeutic modality in brain cancers.

**Figure 4:**
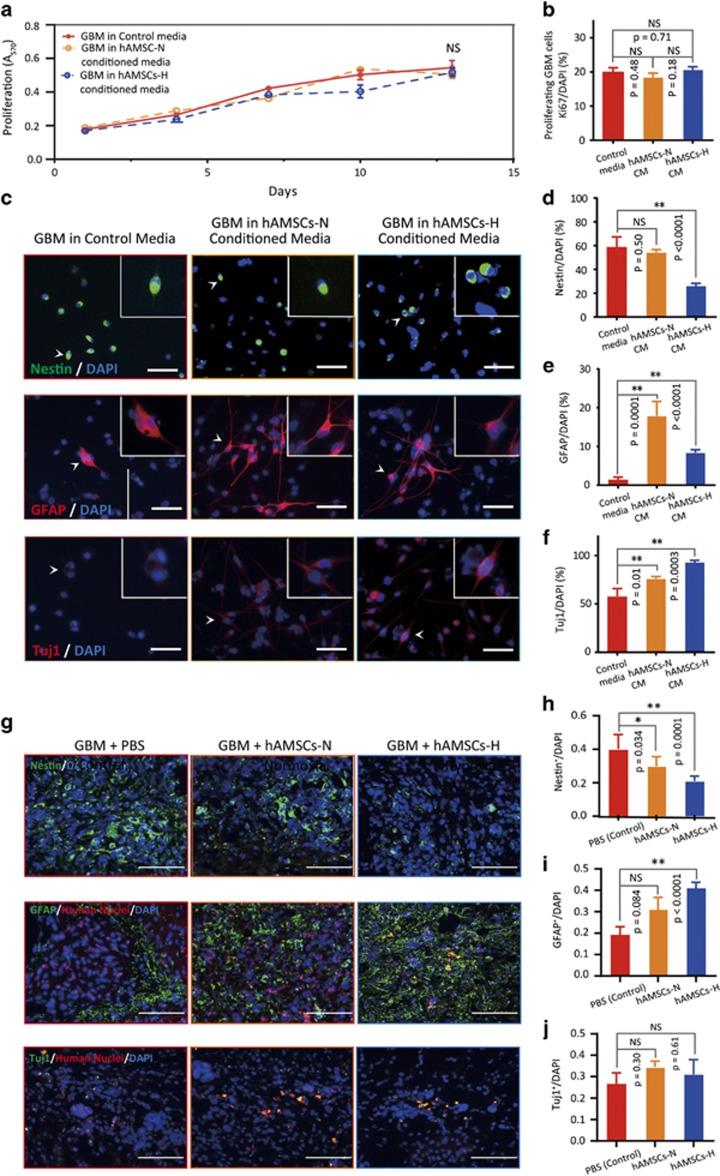
The hAMSC media promotes GBM differentiation *in vitro.* The proliferation and migration abilities of GBM remain unchanged in hAMSC media. *In vivo*, GBM cells exposed to primary hAMSCs-H show increased differentiation into astrocytic lineage (increased GFAP levels and decreased Nestin levels). (**a**) MTT assay was used to determine the effects of hAMSC media on the proliferative capacity of GBM. No difference in GBM proliferative capacity between hAMSC-H or hAMSC-N media and control media was observed. (**b**) Ki-67 immunostaining was performed to quantify the number of proliferating GBM cells in hAMSC media. No difference in proportion of proliferating GBM cells was found between control media, hAMSC-N CM, and hAMSC-H CM. (**c**) Immunostaining for Nestin, GFAP, and Tuj1 was performed on GBM cells in AMSC CM. (**d**–**f**) Quantification of Nestin, GFAP, and Tuj1 markers for GBM cells in hAMSC CM. GBM showed greater differentiation in hAMSC-H CM than in control media as shown by decreased nestin, increased GFAP, and increased Tuj1 expression. (**g**) Mice brain sections were immunostained for Nestin, GFAP, and Tuj1, to test the differentiation of GBM276 cells *in vivo*. GBM276 cells in mice injected with hAMSCs-H showed greater differentiation toward an astrocytic lineage compared with those in mice injected with hAMSCs-N. Scale bars, 100 *μ*m. (**h**–**j**) Quantification of GFAP^+^/human nuclei^+^, Nestin^+^, GFAP^+^, and Tuj1^+^ cells

To confirm the safety of hAMSCs-H and hAMSCs-N CM, we examined GBM proliferation, differentiation, and migration in hAMSC CM *in vitro*. We found no difference in proliferative capacity between GBM cells grown in GBM control media, hAMSC-N CM, or hAMSC-H CM via MTT assay (Figure 4a). There was no difference in the proportion of proliferative GBM cells cultured in GBM control media, hAMSC-N CM, and hAMSC-H CM via Ki-67 staining (Figure 4b). Immunostaining for Nestin, GFAP, and Tuj1 showed decreased Nestin staining (*P*<0.0001), increased GFAP staining (*P*<0.0001), and increased Tuj1 staining (*P*=0.0003), respectively, in GBM cells cultured in hAMSC-H CM compared with GBM complete media, suggesting increased differentiation of GBM cells toward neuronal and astroytic lineages (Figures 4c and f). There was no difference with respect to migration characteristics between GBM cells cultured in GBM control media and hAMSC-N or hAMSC-H CM (Supplementary Figure 6).

In addition, to assess for the effect of hAMSC injection on the differentiation of GBM cells *in vivo*, we performed immunostaining for Nestin, GFAP/human nuclei, and Tuj1 on perfused brain sections 3 weeks after hAMSCs were administered to human GBM-bearing mice (Figure 4g). When injected, primary hAMSCs-H induced greater differentiation of GBM cells into astrocytic lineages compared with control (phosphate-buffered saline (PBS) injection) as evidenced by decreased expression of Nestin (*P*=0.0001) and increased expression of GFAP (*P*<0.0001; Figures 4g–j).

### Primary hAMSCs cultured in hypoxia are not tumorigenic and do not transform into TAFs *in vitro* and/or *in vivo*

**Figure 5:**
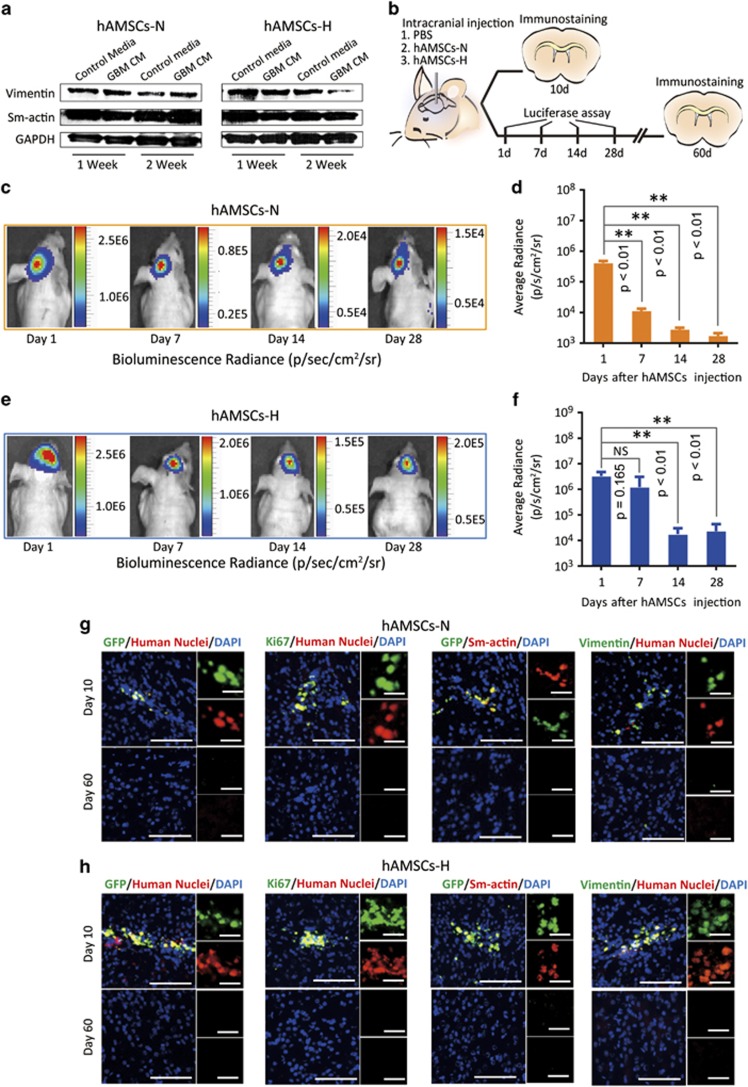
Primary hAMSCs are not tumorigenic and do not transform into TAFs *in vitro* or *in vivo*. (**a**) hAMSCs were cultured in GBM CM or control media for 2 weeks and western blottings (GAPDH served as a control) were performed to quantify TAF markers (vimentin and sm-actin). (**b**) Schematic of the experiment where PBS (*n*=6), hAMSCs-H (*n*=7), or hAMSCs-N (*n*=8) (both of hAMSCs groups labeled with GFP/Luciferase) were injected into mice. The mice (*n*=3 in each group) were separated and killed at day 10. Bioluminescence for the rest of mice in each group was checked on day 1, 7, 14, and 28, then killed at day 60. (**c**–**f**) Live animal imaging of hAMSCs. Bioluminescent radiance was maintained between day 1 and day 7 for hAMSCs-H (*P*=0.165), whereas a significant decrease occurred in hAMSCs-N (*P*<0.01). (**g** and **h**) Vimentin, sm-actin, GFP, and human nuclear stains for hypoxia and normoxia groups. No hAMSCs were seen at day 60 either in hAMSCs-N or hAMSCs-H group. No positive stains for hAMSC markers (GFP/Ki-67 with human nuclei) or TAF markers (vimentin and sm-actin) were observed on day 60. Error bars represent SEM. *P<0.05, **P<0.01, N.S., not significant

As hAMSCs appear to respond favorably to hypoxic conditions, it is critical that they do not become tumorigenic in hypoxia. Therefore, the potential of hAMSCs to undergo malignant transformation into TAFs when exposed to GBM CM was assessed by determining the expression of vimentin and alpha sm-actin, both proteins that are highly expressed in TAFs. Western staining for vimentin and *α*-sm-actin was performed on cell lysates after hAMSCs-N and hAMSCs-H were cultured in GBM CM for 1 and 2 weeks (Figure 5a). Analysis of the intensity of bands relative to GAPDH control showed that hAMSCs-H had decreased vimentin levels and hAMSCs-N had unchanged vimentin levels after 2 weeks of culture in GBM CM. Both hAMSCs-H and hAMSCs-N experienced no change in *α*-sm-actin levels after 2 weeks of culture in GBM CM (Figure 5a). There was no difference in the *α*-sm-actin levels of hAMSCs-H and hAMSCs-N cultured in GBM CM compared with those cultured in control media at 1- and 2-week time points. hAMSCs-H cultured in GBM CM did exhibit a significant decrease in vimentin expression at 2 weeks but not 1 week compared with hAMSCs-H in control media. hAMSCs-N, however, showed no difference in vimentin levels at both time points in GBM CM *versus* control media. These results indicated that neither hAMSCs-N nor hAMSCs-H undergo transformation into TAF-like cells in GBM CM over this time period.

To confirm that hAMSCs-H and hAMSCs-N do not form tumors or TAFs *in vivo*, the safety, proliferative capacity, and survival time of hAMSCs were examined after intracranial injection of either PBS (*n*=6), 0.5 × 10^6^ of GFP/Luciferase transduced hAMSCs-H (*n*=7), or 0.5 × 10^6^ of GFP/Luciferase transduced hAMSCs-*N* (*n*=8). First, we assessed the survival duration of hAMSCs *in vivo*. Mice injected with GFP/Luciferase hAMSCs were imaged 1, 7, 14, and 28 days post injection (schematic shown in Figure 5b). The bioluminescent signal radiance was maintained from day 1 to day 7 in the hypoxia group (*P*=0.165), but there was a significant decrease in the normoxia group (*P*<0.01; Figures 5c–f). These data were consistent with the *in vitro* results, which showed a greater bioluminescence signal in hAMSCs-H compared with hAMSCs-N after 24Mh of culture (Supplementary Figure 7). This implies that hAMSCs-H survive longer than hAMSCs-N *in vivo*. Second, we confirmed that hAMSCs-H and hAMSCs-N do not form tumors or TAFs *in vivo*. Mice were injected with either GFP/Luciferase transduced hAMSCs-H (*n*=3) or hAMSCs-N (*n*=3) and immunostained for GFP, human nuclei, and TAF markers on days 10 and 60. In both hAMSC-H and hAMSC-N injection groups, positive staining for GFP/Ki-67 with human nuclei was observed at day 10 but not on day 60, implying the absence of hAMSCs (Figures 5g and h). In addition, we stained for TAF markers (vimentin and sm-actin) at both time points (Figures 5g and h). In hAMSC-H and hAMSC-N groups, although we observed a positive signal for vimentin and sm-actin on day 10 consistent with normal levels present in human hAMSCs, there was no signal on day 60, implying the absence of both hAMSCs and TAFs. When injected into mice with orthotopic human GBM, neither hAMSCs-N nor hAMSCs-H had any increased expression of Ki-67, TNF-*α*, or VEGF *in vivo,* confirming that they did not undergo malignant transformation in the presence of GBM (Supplementary Figure 8). Staining for expression of vimentin and sm-actin was not performed in hAMSCs injected into mice with orthotopic GBM, as they are endogenously expressed cytoskeletal proteins present in all hAMSCs and would result in a positive staining of all hAMSCs. Thus, we used staining of TNF-*α* and VEGF as alternative markers for TAFs, as their expression levels are significantly changed during cell differentiation, as previously published.^30^

### hAMSCs cultured in hypoxia have enhanced migration and tropism toward brain cancer *in vitro* and *in vivo*

**Figure 6:**
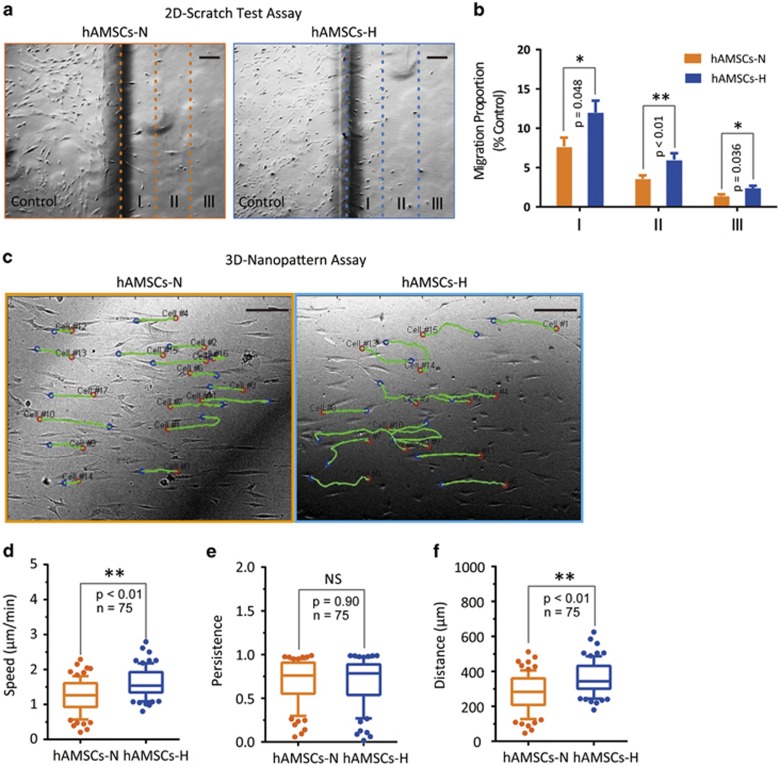
Hypoxia enhances migration ability of primary hAMSCs. (**a**) 2D-Scratch test. The plates with different groups of hAMSCs were scratched for 5 mm margin space and the borderline of the scratch was marked immediately. Then, the photographs were captured with bright-field microscopy at the endpoint of 24 h (scale bar, 200 *μ*m). (**b**) Comparison of migration proportion between hAMSCs-H and hAMSCs-N in zones I, II, and III. (**c**) Representative tracks of hAMSCs-H and hAMSCs-N group. Cell migration was quantified using time-lapse microscopy on 3D nanopattern surface. Images were automatically recorded for 6 h at 10-min intervals. (**d**–**f**) Speed, persistence, and distance were assessed by tracking 75 cells in each group. Error bars represent S.E.M. *P<0.05, **P<0.01, N.S., not significant

We further investigated the long-term effects of hypoxia culture conditions on hAMSC migration and tropism toward brain cancer, both *in vitro* and *in vivo*. The effects of hypoxia on hAMSC migration were assessed using the two-dimensional scratch test and a 3D nanopattern migration assay *in vitro*. On the scratch test, hAMSCs-H had a significantly greater migratory capacity than hAMSCs-N (Figures 6a and b). For the 3D nanopattern model with nano-grooves for cell alignment (Figure 6c), hAMSCs-H had a significantly greater migration speed (*P*<0.01) and traveled distance (*P*<0.01) than hAMSCs-N (Figures 6d–f and Supplementary Video 1). Similarly, increases in migratory capacity using the 2D scratch assay (Supplementary Figures 9A and B), migration speed (Supplementary Figure 9D and Supplementary Video 2) and traveled distance (Supplementary Figure 9F) in the 3D-nanopattern model were observed in commercial hAMSCs-H.

**Figure 7:**
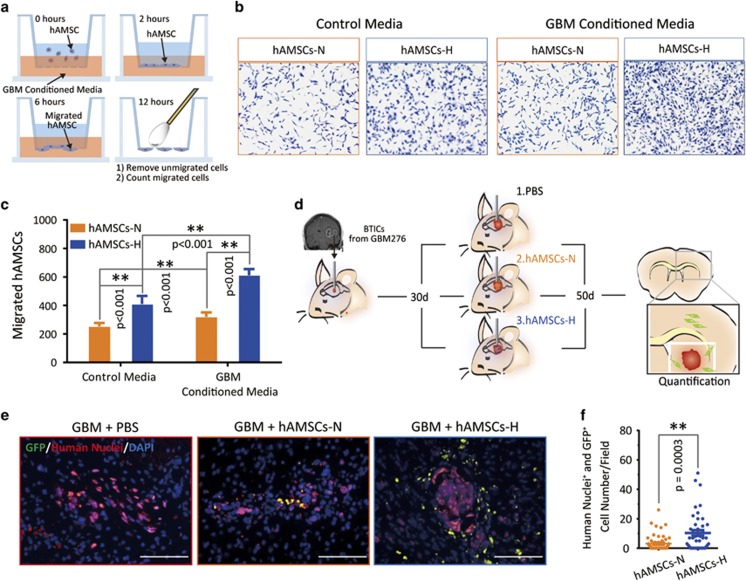
The tropism of primary hAMSCs-H to GBM CM *in vitro* and GBM *in vivo* is increased. (**a**) Schematic of a Boyden chamber transwell assay. (**b** and **c**) Representative images and quantification graphs shown for a Boyden chamber transwell assay. hAMSCs (2 × 10^4^) were seeded in the top well, while either GBM CM or control media was placed in the bottom well. After 24-h incubation, cells on the bottom were stained and quantified. (**d**) Schematic of the *in vivo* experiment. (**e**) Mice brain sections were immunostained for GFP and human nuclei to test the tropism of the primary hAMSCs for GBM276 tumor bulk *in vivo*. hAMSCs-H have enhanced tropism toward tumor bulk compared with hAMSCs-N. Scale bars, 100 *μ*m. (**f**) Quantification of percentage of GFP^+^/human nuclei^+^ cells per field. Error bars represent S.E.M. *P<0.05, **P<0.01, N.S., not significant

To assess the cancer tropism of hAMSCs, we performed *in vitro* Boyden transwell chamber assays (schematic, Figure 7a), which demonstrated significantly greater migration of hAMSCs-H toward GBM CM (collection described in schematic, Figure 3b) compared with hAMSCs-N (*P*<0.001) (Figures 7b and c). Similar results were seen with commercial hAMSCs (Supplementary Figures 10A and B).

We further evaluated, *in vivo*, the effect of hypoxic culture conditions on hAMSC tropism toward GBM cells. GFP-hAMSCs (0.5 × 10^6^ cells/mouse) were injected into the striatum of athymic mouse 30 days after GBM276 brain tumor-initiating cells (BTICs; 0.3 × 10^6^ cells/mouse) were injected into the ipsilateral deep striatum (schematic, Figure 7d). The decision to inject GFP-hAMSCs into the ipsilateral deep striatum *versus* the contralateral side were made after preliminary studies (not shown) using contralateral deep striatum injection showed that hAMSCs could not successfully migrate to the contralateral striatum during the time window for implantation of brain cancer and animal survival. To exclude the possibility that GFP-hAMSCs could have been administered into the same injection trajectory previously used for GBM276 BTIC injection, resulting in a spurious finding of hAMSCs around the tumor bulk, cryosectioning and immunostaining of mice brains with GFP/human nuclei near the tumor bulk 1 day after GFP-hAMSC injection was performed, showing no hAMSCs near the tumor bulk at this early time point (data not shown). After 20 days, the mice were killed and their brains cryosectioned and immunostained with GFP/human nuclei near the tumor bulk, to identify hAMSC tropism toward the tumor (Figure 7e). There were a greater quantity of hAMSCs-H per visual field near tumor than hAMSCs-N (*P*=0.0003) (Figure 7f). Although the enhanced migration (Figures 6a and d) and tumor tropism (Figures 7a and c) *in vitro* may support the explanation that this result is an effect of hAMSCs-H's enhanced tropism toward GBM, it is also possible that this finding of a greater quantity of hAMSCs-H near the tumor than hAMSCs-N could have been due to the increased survival of hAMSCs-H *in vivo* (Figures 5b–f), as they were injected ipsilateral to the tumor bulk.

## Discussion

Patients with brain cancer are afflicted with a disease process that is able to escape local therapy.^12, 36, 37^ Individual cells within brain cancer, namely GBM, are able to migrate from the tumor bulk and invade surrounding parenchyma, making local therapies such as surgical resection and radiation therapy difficult.^12, 36, 37^ An ideal treatment option would be one that involves targeting these individual migrating brain cancer cells.^38, 39, 40, 41^ Interestingly, MSCs not only possess the ability to home to cancer cells,^20, 30, 42^ but they can also be engineered to serve as therapeutic delivery vehicles^30, 43^ These collective abilities make MSCs an attractive potential treatment option for not only brain cancer, but a wide variety of other neurological diseases including stroke, Parkinson's disease, and multiple sclerosis. Sources of MSCs include the bone marrow, adipose tissue, and muscle. Regardless of source, MSCs need to be expanded in culture, because the number of cells collected from these sources is too small to have a therapeutic effect. This study found that under hypoxic conditions, primary, intra-operatively derived hAMSCs are more stable in cell culture, have an increased proliferative capacity, retain their multipotency, are non-tumorigenic, and have an enhanced tropism to GBM *in vivo.* These hypoxic conditions are more analogous to their *in vivo* environments from which they are derived.

A major obstacle for stem cell therapeutic use is their low collection rate from tissues. MSCs, for example, account for as little as 0.001–0.01% of bone marrow tissue^44^ and up to only as much as 1.2–5.1% of the stromal vascular fraction of adipose tissue.^22^ Therefore, regardless of source, a prerequisite to stem cell therapeutic use is *in vitro* expansion. Most stem cell culturing conditions occur in normoxic conditions at 21% oxygen tension, while most body tissues including adipose has a physiological oxygen tension of<3%.^45^ Ni *et al.*^46^ studied the effects of hypoxia on human placental MSCs and found that low oxygen tensions increased the proliferation of these placenta-derived MSCs. Likewise, Sugure *et al.*^47^ found that hypoxia not only increased the proliferation of BM-MSCs, but also increased their survival. Our study also found that hypoxic conditions promote proliferation and survival, as well as the enhanced migratory ability of primary hAMSCs. These effects are believed to be mediated via hypoxia-inducible factor-1*α*, which is unregulated in the presence of hypoxic conditions.^48, 49, 50^

In order for hypoxia-cultured MSCs to be used clinically for brain cancer, their tumor tropism must be maintained. Previous studies have not yet established whether hypoxia affects their migratory ability. In this study, we found that hypoxia actually promotes hAMSC migration toward cancer cells, in both *in vitro* and *in vivo* conditions. Importantly, hypoxia, despite promoting proliferation, should not cause these cells to undergo oncogenic transformation. We did demonstrate in a previous study that hAMSCs do not form TAFs or undergo oncogenic transformation when exposed to brain cancer,^30^ *in vivo*, but the effect of hypoxia on these findings had not yet been elucidated. The current study demonstrates that hypoxia does not increase the expression of TAFs, and that hAMSCs do not undergo oncogenic transformation *in vivo*, even in the presence of brain tumor. Furthermore, in our model of GBM, hAMSCs did not show an increase in the expression of Ki-67 or markers of tumor growth (TNF-*α* and VEGF). These results suggest that hAMSCs are neither tumor supportive nor tumorigenic. This is consistent with our previous study, which found that the injection of hAMSCs significantly decreased tumor size and caused a trend toward increased survival in a murine model of human GBM.^30^ These features are critical for the efficacy of hypoxia in expanding these cells *ex vivo*.

We found that hAMSCs may promote the differentiation of GBM cells into astrocytic lineages, both *in vitro* and *in vivo*. This is important, as the primary GBM cell line 276 used in this study belongs to the mesenchymal subtype, which is the most aggressive subtype.^51^ The hypoxia-cultured hAMSCs had enhanced tropism and attenuated the malignant tumor characteristics of GBM276 used in this study. Moreover, we found that hAMSCs decreased the expression of the angiogenesis marker VEGF *in vivo*. Therefore, hAMSCs may contribute to better outcomes in GBM via multiple pathways, of which angiogenesis is one pathway. All these outcomes imply the utility of hAMSCs-H as vehicles for the delivery of treatment for brain cancer.

Although we did find encouraging data that showed that hAMSCs-H have higher migration toward brain tumor *in vitro* (Figure 7c), display greater tropism toward human brain tumor *in vivo* (Figures 7e and f), survive longer in the brain *in vivo* (Figures 5c and f), and promote tumor cell differentiation (Figures 4c–j), several limitations must be taken into account regarding the implications of this data. hAMSCs did not affect brain tumor cell proliferation (Figures 4a and b), *in vivo* Ki-67 (proliferative marker) expression in GBM (Supplementary Figure 8), or GBM expression of TNF-*α*, a necrosis marker (Supplementary Figure 8). Thus, unmodified hAMSCs alone may have limited therapeutic potential for brain cancer. Rather, the approach of using hAMSCs as therapeutic delivery vehicles for anti-cancer agents such as BMP4 (Li *et al.*^30^) may increase the ability of hAMSCs to inhibit brain tumor cell proliferation and malignancy while taking advantage of their innate tumor tropic abilities. Besides brain cancer, MSCs are also being studied for their ability to replace damaged cells in a variety of organs including the ischemic cells following stroke, cartilage in aging, damaged neurons after traumatic brain injury, and osteoblasts in osteoporotic patients, among others.^45, 52, 53, 54, 55^ Our study found that hypoxia allows hAMSCs to retain the ability to differentiate into a variety of cell lineages including osteocytes, adipocytes, and chondrocytes. Hypoxia does not preclude these cells from differentiating into different mesenchymal lineages.

We demonstrate that hypoxia has several potential useful effects on hAMSCs. First, hypoxia promotes the proliferation and survival of primary intraoperatively obtained hAMSCs. Second, despite this increase in proliferation of hAMSCs, it does not promote oncogenic transformation or the formation of TAFs. Third, hypoxia potentiates the tumor-suppressive ability of hAMSCs to cause differentiation of brain cancer cells. Lastly, hypoxia increases the ability of hAMSCs to migrate and home to brain cancer cells. Taken together, hypoxia can potentially increase the therapeutic effect of hAMSCs not only for brain cancer, but for other pathologies as well. Future research should attempt to elucidate the bidirectional regulation between hAMSCs and tumor cells mediating the ability of hAMSCs to migrate toward and/or suppress brain cancer cells. In breast cancer, it has been reported that bidirectional paracrine signaling between BM-MSCs and breast cancer cells promotes the migration of MSCs toward cancer cells via hypoxia-inducible factor-dependent pathways.^56^ It is not known whether the same is true between hAMSCs and brain cancer.

## Materials and Methods

### Cell cultures

#### Primary hAMSC culture (patient derived)

Abdominal adipose tissue was obtained intraoperatively from patients undergoing surgery (as approved by the Johns Hopkins Institutional Review Board). Adipose tissue was transferred to Hank's balanced salt solution (Cellpro, Bothell, WA, USA; 21-022-CV) and processed within 3 h. To culture the primary cells, every 4 g of tissue was digested with 4 ml of 1 mg/ml collagenase A (Roche, San Francisco, CA, USA; 10103 578 001) for 20 min at 37 °C, with manual mixing every 5 min. Collagenase activity was inhibited by adding an equal volume of MesenPRO complete media (1% Antibiotic/Antimycotic (Invitrogen, Waltham, MA, USA; 15240-062), 1% Glutamax (GIBCO, Grand Island, NY, USA; 35050-061), one vial of MesenPRO RS growth supplement (GIBCO, 12748-018) and MesenPRO RS basal media, (GIBCO, 12747-010), and then centrifuged at 300 × *g* for 10 min at 4 °C. The cell pellet obtained from 4 g tissue was re-suspended in 4 ml MesenPRO complete media, seeded in two 35-mm petri dishes, and maintained in hypoxic (1.5% O_2_) (Thermo, Waltham, MA, USA; Heracell 150i) or normoxic (21.0% O_2_) incubators (Thermo, Forma Series II) (Table 1). Cultures were kept under 80% confluence and passaged when they reached this confluency, to avoid contact inhibition. Primary hAMSC lines were frozen at passage 2. Unless specified otherwise, primary hAMSC line 1101 was used for all experiments and all experiments were started 10 days after thawing.

#### Commercial hAMSC culture

Commercial hAMSCs (Invitrogen, R7788-115) were cultured in MesenPRO complete media as described above. Cultures were kept under 80% confluence to avoid contact inhibition and passaged when they reached this confluency. Commercial hAMSCs were frozen at passage 3. All experiments on commercial hAMSCs were started 10 days after the cell culture was thawed from its frozen stock.

#### Brain tumor-initiating cell culture

BTICs derived from a human primary GBM (GBM 276, mesenchymal type) were also obtained from intraoperative tissue (as approved by Johns Hopkins Institutional Review Board) and cultured in laminin-coated flasks (Sigma, St. Louis, MO, USA; L2020, 1 *μ*g/cm^2^) using complete stem cell media with 96% DMEM/F12 (Invitrogen, 11330-032), 2% Antibiotic/Antimycotic (Sigma, A5955), 2% B27 (Invitrogen, 17504-044), 20 ng/ml hFGF-b (PeproTech, Rocky Hill, NJ, USA; 100-18B), and 20 ng/ml hEGF (PeproTech, AF-100-15)). GBM 276 has been previously validated and forms tumors when implanted into mice as previously shown by our group.^38^ In addition, a metagene score-based approach for subtype designation assessing four mesenchymal and two proneural genes using a microfluidics-based quantitative PCR assay was performed to identify the mesenchymal molecular subtype of GBM 276 as previously described.^57^ GBM 276 was maintained in a normoxic incubator at 37 °C and an atmosphere of 5% CO_2_.^30, 39^

### Media collection

To collect soluble factors released by GBM 276 BTICs, 5 × 10^5^ cells/well were seeded in six-well plates and cultured in MesenPRO complete media (Table 2). This media was conditioned for 24 h and then collected and passed through a 0.45-*μ*m filter (Corning, Corning, NY, USA; 431220). This media was stored at −80 °C and thawed before use. Any remaining GBM CM was stored at 4 °C and used within a week of thawing. To collect soluble factors released by hAMSCs-H and hAMSCs-N, a similar protocol was followed by seeding 5 × 10^5^ cells/well in a six-well plate and culturing in GBM complete media (Table 2). This media was conditioned for 24 h, followed by filtering and storing as described above. The formulations of all media described in this paper are summarized in Table 2.

### Stemness analysis

#### Differentiation assay for hAMSCs

To determine the differentiation capacity of hAMSCs, cells were seeded on 24-well plates (42 000 cells/well for adipogenic differentiation and 8400 cells/well for osteogenic differentiation), or cultured as pellets (250 000 cells/tube for chondrogenic differentiation). Cells were cultured in differentiation media as specified by the manufacturer (R&D, Minneapolis, MN, USA; CCM005-008, and CCM011), MesenPRO complete media without differentiation supplements, and GBM CM for 21 days. Cell lineage was evaluated using Oil Red O (Sigma, 00625), Alizarin Red S (Sigma, AB5533), and Masson's Trichrome, which stained for adipocytes, osteocytes, and chondrocytes, respectively.

#### hAMSC marker analysis

hAMSC phenotype was confirmed by flow cytometric analysis according to the criteria from the ISCT^1, 2^ and as we have previously reported.^20, 30^ Monoclonal antibodies (CD31-FITC 11-0319, CD34-FITC 11-0341, CD45-FITC 11-0459, CD73-APC 17-0739, CD90-PeCy5 15-0909, and CD105-PE 12-1057) from eBioscience (San Diego, CA, USA) were used. hAMSCs (7 × 10^5^) were trypsinized and re-suspended in 7 ml blocking solution (1% BSA/PBS with 10% FBS) for 10 min. For each staining, 1 ml aliquots were centrifuged at 300 × *g* for 5 min, re-suspended in 100 *μ*l buffer (1% BSA/PBS), stained with 5 *μ*l primary-conjugated antibodies, and then incubated for 30 min. Cells were washed with PBS twice, then tested using a four-channel flow cytometer (FacsCalibur, Beckman Coulter, Hialeah, FL, USA) to acquire 20 000 events for analysis.

#### Cell morphology analysis on a 3D nanopattern surface

The morphology of cells was analyzed using a nanopatterned 3D surface (ridge 350 mm, groove 350 nm, height 150 nm). This 3D nanopattern has nano-ridges and grooves constructed of transparent poly urethane acrylate and is fabricated using UV-assisted capillary lithography as previously reported by our group.^30, 39^ Cells were placed on the surface and imaged under an inverted microscope (AxioObserver Z1, Zeiss, Richmond, VA, USA) as previously reported by our group.^30, 39^ Nanopattern surfaces were coated with laminin (5 *μ*g/cm^2^) and 20 000/well of hAMSCs were plated in MesenPRO complete media. The length, width, and area of 40 cells in each group were measured using Axiovison version 4.8 (Zeiss).

### Proliferation and viability analysis

#### Passage time of P0, P1, and P2

The passage time P0 for primary cell cultures was calculated from fat tissue processing day 0 to the day when cells from 2 g of tissue reached 80% confluence in a 35-mm petri dish. Cells were consequently digested and seeded into a T25 flask at the density of 0.6 × 10^4^/cm^2^. The time to reach 80% confluence was calculated as passage time P1. The cells then underwent trypsinization for a second time and were re-seeded into a new flask at the same density of 0.6 × 10^4^/cm^2^. The time needed to reach 80% confluence was calculated as passage time of P2.

#### PDT assay

hAMSCs were seeded in 12-well plates at the density of 0.5-1 × 10^4^/cm^2^ in triplicates. When they reached 80% confluence, cells were collected and counted. PDT was calculated using the equation: PDT=*τ* Ln (2)/Ln (*N*_*τ*_/*N*_0_), where *τ*=time from plating to counting the cells, *N*_*τ*_=number of cells when counted, and *N*_0_=initial number of cells.

#### *In vitro* hAMSCs proliferation assay

MTT (Sigma, M5655) assay was used to determine the effects of hypoxia on the proliferative capacity of hAMSCs. hAMSCs (1000 cells/well, respectively, at passage 3 for primary hAMSCs and passage 4 for commercial hAMSCs) were seeded in 96-well plates and cultured for 12 days in MesenPRO complete media or GBM CM, which was collected following the ‘Media Collection' protocol above and as previously reported by our group.^20, 30^ In addition, for proliferative analysis of GBM in primary hAMSC CM, MTT assay was used as described above with GBM cells in hAMSC-H CM and hAMSC-N CM. Cell proliferation was analyzed at different time points in triplicates for each experimental condition.

To determine the percentage of proliferating cells, immunostaining for Ki-67 (Leica, Buffalo Grove, IL, USA; NCL-Ki67p) was quantified. hAMSCs (1500 cells/cm^2^) were seeded on glass slides pre-coated with poly-L-ornithine (Sigma, P4957) and laminin within a 24-well plate and cultured in MesenPRO complete media for 2 days. Then, cells were fixed with 4% PFA, blocked with 10% normal goat serum, and immunostained for Ki-67. Alexa-labeled secondary antibodies (Life Technologies, Carlsbad, CA, USA; A11037) and DAPI (Invitrogen, D1306) were used for visualization of markers and cell nuclei, respectively. Images were visualized and captured with an inverted fluorescence microscope (AxioObserver Z1, Zeiss). The number of Ki-67-positive cells was counted from 10 to 15 random fields by blinded observers. In addition, for proliferative analysis of GBM in primary hAMSC CM, Ki-67 staining was used as described 2 days after GBM cells (1500 cells/cm^2^) were seeded on glass slides pre-coated with poly-L-ornithine (Sigma, P4957) and laminin within a 24-well plate and cultured in hAMSC-H or hAMSC-N CM.

#### hAMSCs apoptosis and cell cycle assay

Flow cytometric analysis was performed using an Annexin V-fluorescein isothiocyanate (Annexin V–FITC) apoptosis antibody (BD Pharmingen, San Diego, CA, USA; 556419), according to the manufacturer's instructions. Briefly, cells were collected and re-suspended in binding buffer. Annexin V–FITC and PI were added, and the reaction was incubated in the dark for 15 min. Cells were analyzed using a FACScan flow cytometer. Annexin V–FITC and PI analysis was performed in triplicate for each experimental condition.

PI staining was used to test the cell cycle and necrosis percentage of the cells. Cells were collected and fixed with 100% ethanol (kept in −20 °C) added drop-wise while gently vortexing. The fixation reaction was incubated for 24 h at 4 °C. Cells were then centrifuged and re-suspended in PBS containing 0.1% Triton X-100 and 10 *μ*g/ml RNase, and incubated for 20–30 min at 37 °C. PI was added at a final concentration of 40 *μ*g/ml and cells were analyzed using flow cytometry.

### Safety and tumorigenic analysis

#### Western blot assay

hAMSCs were cultured in GBM CM or MesenPRO complete media for 1 or 2 weeks, then total protein was isolated from samples with RIPA buffer (Thermo, 89901) to quantify TAF markers vimentin (Abcam, San Francisco, CA, USA; ab16700) and *α*-sm-actin (Abcam, ab54723).

#### Lentiviral production and infection

To identify hAMSCs in our *in vivo* mouse experiments, we transduced primary cultured P1 cells with lentiviral vectors coding for GFP/luciferase proteins. Viral vectors were packaged from HEK293 cells. After collection and concentration, hAMSCs were sorted by flow cytometry to select GFP/luciferase-expressing cells.

### Motility and tropism analysis

#### 2D scratch assay

hAMSCs were grown to 50–60% confluence and then the plates were scratched with a sterile cell scraper (Fisher, Waltham, MA, USA; 08-773-2) to generate a 5-mm-wide area free of cells. The scratch border was marked with a fine line immediately after the scraping. The migration of cells was assessed as a function of how far from the scratch line the cells had migrated to the margin place over the course of 12 h. The cells were then fixed with 4% PFA and stained with DAPI (Invitrogen, D1306) for visualization of cell nuclei. The number of nuclei that had migrated over the scratch line was counted using the Image J program version 1.47 (NIH, Bethesda, MD, USA) (more than 12 random fields in each group were counted by blinded observers).

#### 3D nanopattern migration assay

hAMSCs were placed on the surface of a 3D nanopattern as described in ‘Stemness analysis: Cell morphology analysis on a 3D nanopattern surface.' Cell migration was quantified using time-lapse microscopy. Long-term observation was performed with a motorized inverted microscope (Olympus IX81, Olympus America, Center Valley, PA, USA). Phase-contrast and DAPI-fluorescent cell images were automatically recorded for 6 h at 10-min intervals using the Slidebook 4.1 (Intelligent Imaging Innovations, Denver, CO, USA). Cell speed was calculated based on blinded observers tracking 75 cells per condition and using customized semi-automated program developed with MATLAB version 2008 (Natick, MA, USA).

#### *In vitro* tropism transwell assay

Cell migration was evaluated using the Boyden chamber (Corning, 3422) assays as previously reported by our group.^30^ To quantify hAMSC tropism to GBM CM *in vitro*, 2 × 10^4^ hAMSCs in 100 *μ*l MesenPRO complete media were seeded in the top well of the chamber, while either 600 *μ*l GBM CM or MesenPRO complete media were placed in the bottom well. After 12-h incubation, cells on top of the membrane were removed using cotton swabs and cells on the bottom were stained with the Diff-Quik stain set (Siemens, Munich, Germany; B4132-1 A) for counting.

### *In vivo* analyses

#### *In vivo* hAMSC luciferase assay and tumorigenic assay

To investigate the safety of GFP/luciferase-hAMSCs *in vivo*, 6- to 8-week-old NOD/SCID mice were randomly divided into three groups and stereotactically injected with 5 × 10^5^ hAMSCs or an equal volume of PBS into the left striatum (AP=−0.8 mm, L=1.5 mm, H=3.0 mm) in the normoxia group or the right striatum (AP=−0.8 mm, R=1.5 mm, H=3.0 mm) in the hypoxia group. Following injection, mice in the GFP/luciferase-hAMSC group were imaged using an IVIS small animal imaging system (PerkinElmer, Waltham, MA, USA; IVIS Spectrum) at different time periods (day 1, day 7, day 14, and day 28). At either day 10 or day 60, animals were euthanized and perfused with 4% PFA. Brains were extracted, cryo-sectioned, and immunostained for GFP (Abcam, ab6662), vimentin, *α*sm-actin and human nuclei (Millipore, Billerica, MA, USA; MAB4383).

### *In vivo* GBM differentiation and malignancy analysis

To determine the differentiation and malignancy of GBM tumor *in vivo*, 0.3 × 10^6^ human GBM 276 BTICs were stereotactically injected into the right striatum (AP=−0.8 mm, H=3.0 mm) of immunosuppressed nude mice. Thirty days post injection, 0.5 × 10^6^ GFP/luciferase-hAMSCs or PBS were stereotactically injected into the brain (AP=−0.8 mm, H=1.5 mm) of the same side. After 3 weeks, the mice were perfused. The perfused mice brain sections were immunostained for stemness marker Nestin (Millipore, MAB5326), astrocytic marker GFAP (DAKO Z0334, Glostrup, Denmark), neural marker Tuj1 (Covance, Princeton, NJ, USA; PRB-435p), proliferation marker Ki-67 (Leica VP-K451), necrosis marker TNF-*α* (Abcam, ab6671), and pro-angiogenic marker VEGF (Abcam ab46154). GBM cells were identified from the background of mouse brain cells by staining for human nuclei (red) and DAPI (blue). Although injected GFP/luciferase-hAMSCs did express GFP, the green signal from GFP fluorescence was significantly weaker than the green signal of immunostaining for Nestin, GFAP, and Tuj1, and therefore did not interfere with the identification of these green fluorescence-marked proteins. Quantification of cells was performed.

### *In vivo* hAMSC tropism study

To determine the tropism capacity of hAMSCs for orthotopic GBM tumor *in vivo*, 0.3 × 10^6^ GBM 276 BTICs were stereotactically injected into the right striatum (AP=−0.8 mm, H=3.0 mm) of immunosuppressed nude mice. Thirty days post injection, 0.5 × 10^6^ GFP/luciferase-hAMSCs or PBS were stereotactically injected into the brain (AP=−0.8 mm, H=1.5 mm) of the same side. After 3 weeks, the mice were perfused, brains extracted, cryo-sectioned, and immunostained for GFP (Abcam, ab6662) for hAMSCs and human nuclei (Millipore, MAB4383) for both hAMSCs and GBM cells. To control for the characteristic of tumor bulk on the tropism of hAMSCs toward tumor, the number of cells in each tumor bulk were counted to assure that tumor sizes were consistent (Supplementary Figure 1). All *in vivo* procedures were approved by the Johns Hopkins University Animal Care and Use Committee.

### Statistical analysis

Results are reported as mean±SEM. Comparisons were done using two-way ANOVA for MTT assays and luciferase assays. Student *t*-tests (Mann–Whitney) were used for other experiments using GraphPad Prism 6 (La Jolla, CA, USA) software. Statistical significance was defined as *P<0.05 and **P<0.01.

## Figures and Tables

**Table 1:** Demographics of non-cancer patients from whom adipose tissue was obtained

**Patient**	**Cell culture**	**Age (years)**	**Gender**
1	1074	66	M
2	1077	47	F
3	1082	42	F
4	1086	52	M
5	1089	37	F
6	1097	33	F
7	1098	73	M
8	1100	49	M
9	1101	41	F

Abbreviations: F, female; M, male

**Table 2:** Formulations of media used in this study

*Complete/control media*	
MesenPRO complete media (for hAMSCs)	MesenPRO RS basal media (GIBCO, 12747-010) 1% Antibiotic/Antimycotic (Invitrogen, 15240-062) 1% Glutamax (GIBCO, 35050-061) 1 vial of MesenPRO RS growth supplement (GIBCO, 12748-018)
GBM complete media (for 276 BTICs)	96% DMEM/F12 (Invitrogen, 11330-032) 2% Antibiotic/Antimycotic (Sigma, A5955) 2% B27 (Invitrogen, 17504-044) 20 ng/ml hFGF-b (PeproTech, 100-18B) 20 ng/ml hEGF (PeproTech, AF-100-15)
	
*Conditioned media*	
hAMSC CM	2.5 × 10^5^ cells per well in a six-well plate and cultured in GBM complete media. After 24 h, the medium was collected and passed through a 0.45-*μ*m filter.
GBM CM	5 × 10^5^ cells per well seeded in six-well plates and cultured in MesenPRO complete media. After 24 h, the medium was collected and passed through a 0.45-*μ*m filter.

Abbreviations: BTIC, brain tumor-initiating cell; CM, conditioned media; hAMSC, human adipose-derived mesenchymal stem cell; lGBM, gliolastoma

## Abbreviations

hAMSC: human adipose-derived MSC
MSC: mesenchymal stem cell
GBM: glioblastoma
BM-MSC: bone marrow-derived MSC
TAF: tumor-associated fibroblast
BTIC: brain tumor-initiating cell
ISCT: International Society for Cellular Therapy
