# Supplementary figures and images for: Hypoxia-cultured human adipose-derived mesenchymal stem cells are non-oncogenic and have enhanced viability, motility, and tropism to brain cancer
Source: Cell Death Dis. 2014 Dec 11;5(12):e1567–. doi: 10.1038/cddis.2014.521 (PMC4649837; doi:10.1038/cddis.2014.521)

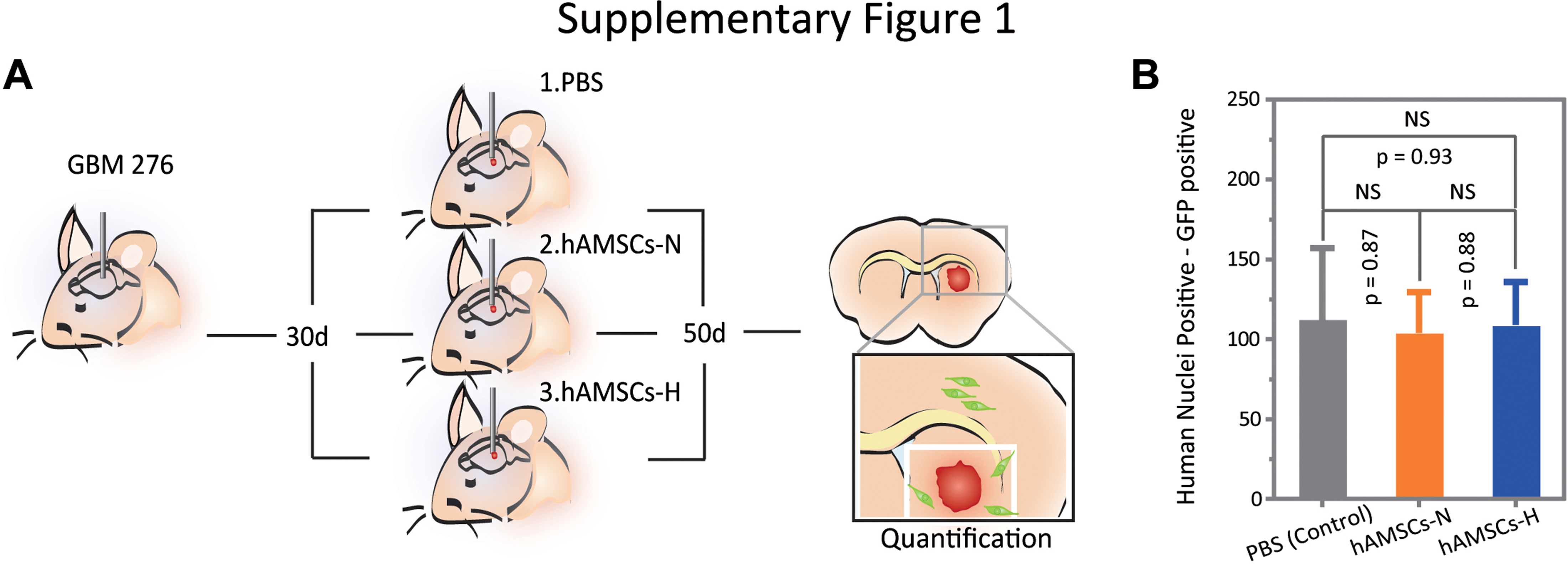

Supplement: Supplementary Figure 1 [file cddis2014521x1.tif]

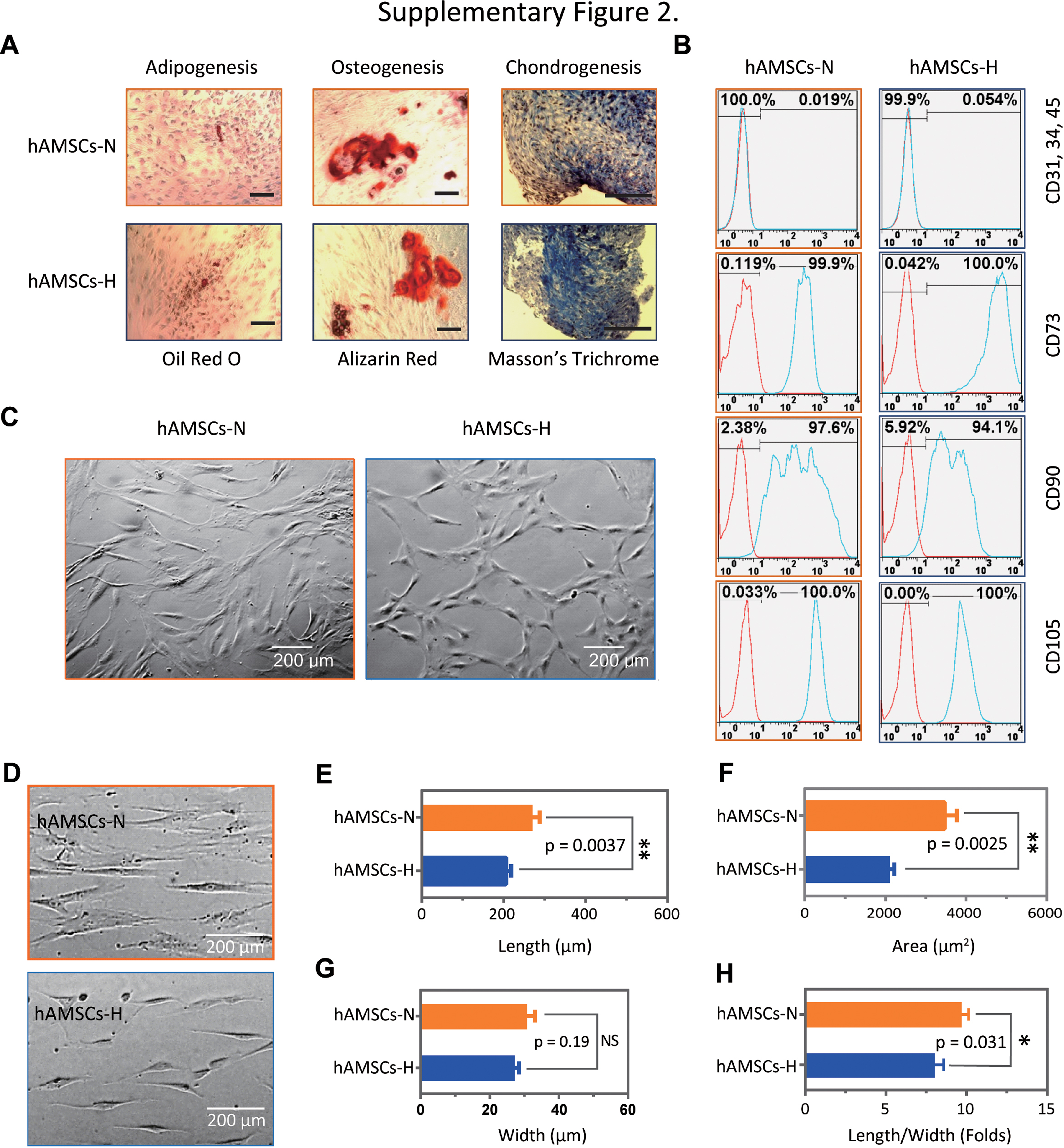

Supplement: Supplementary Figure 2 [file cddis2014521x2.tif]

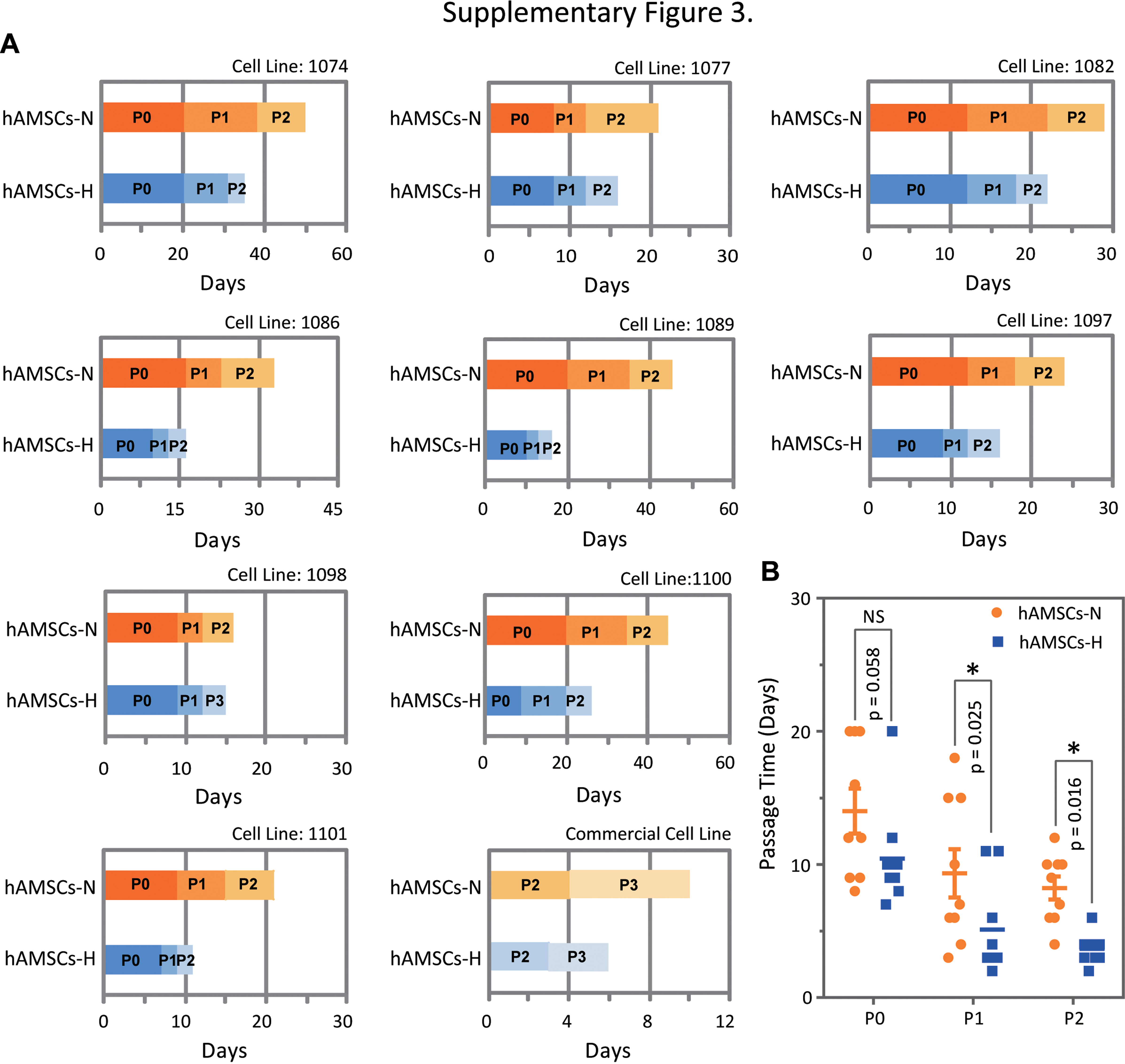

Supplement: Supplementary Figure 3 [file cddis2014521x3.tif]

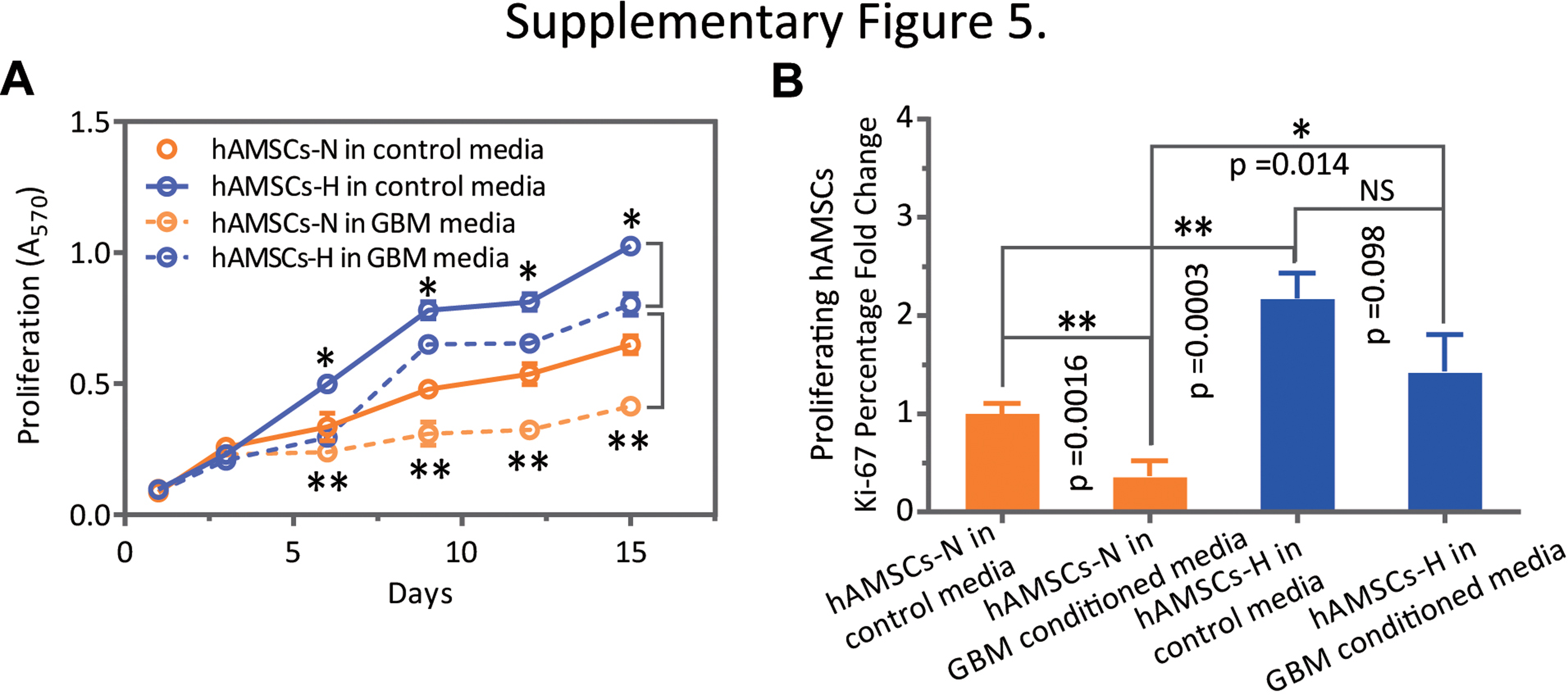

Supplement: Supplementary Figure 4 [file cddis2014521x4.tif]

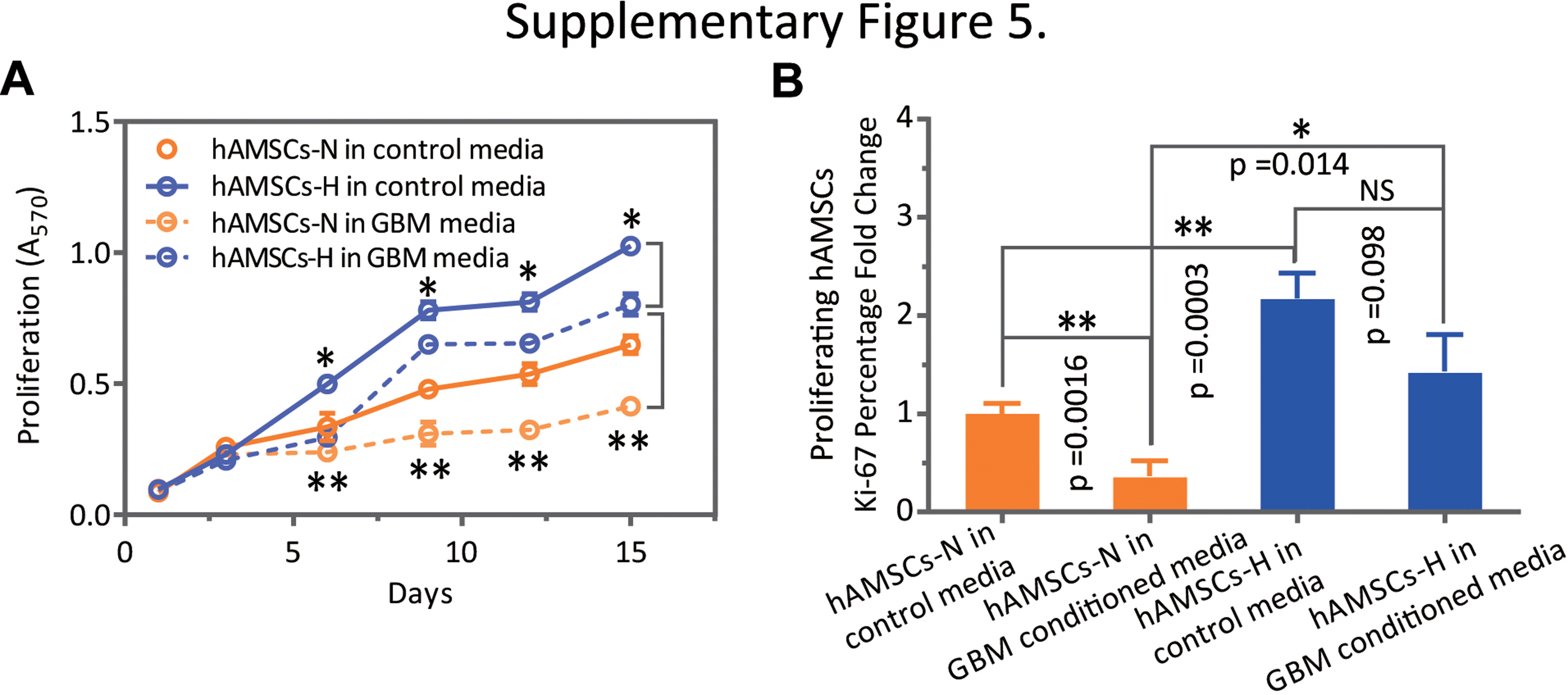

Supplement: Supplementary Figure 5 [file cddis2014521x5.tif]

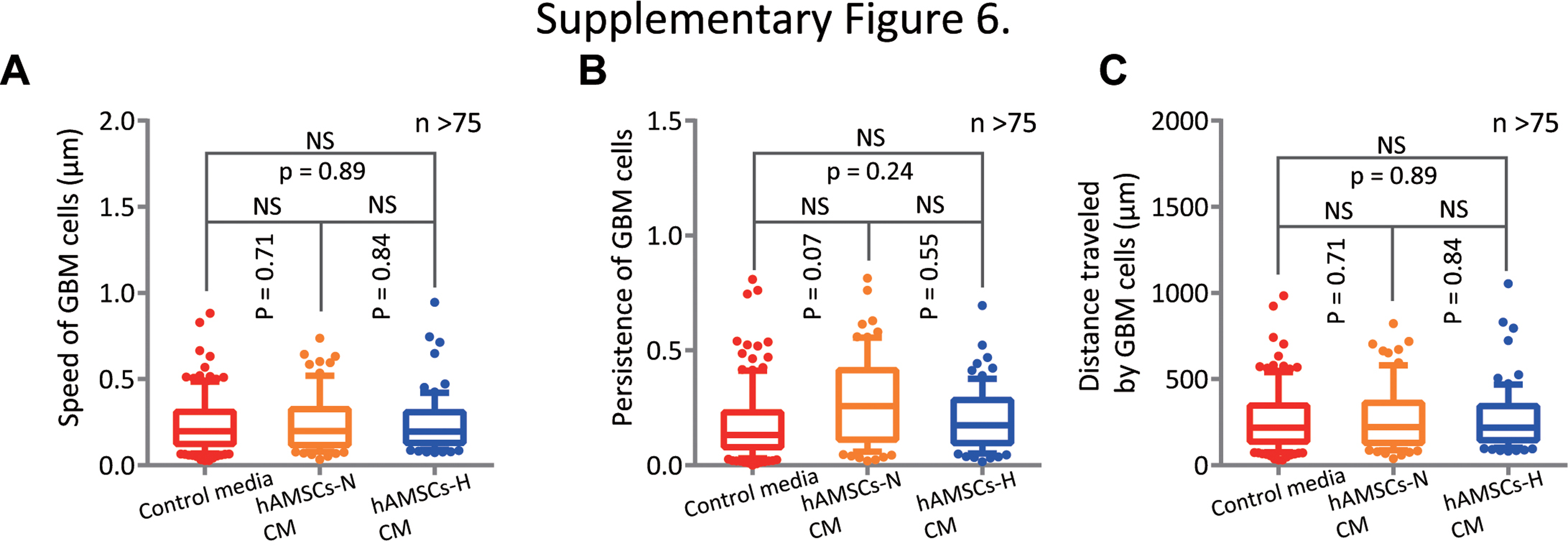

Supplement: Supplementary Figure 6 [file cddis2014521x6.tif]

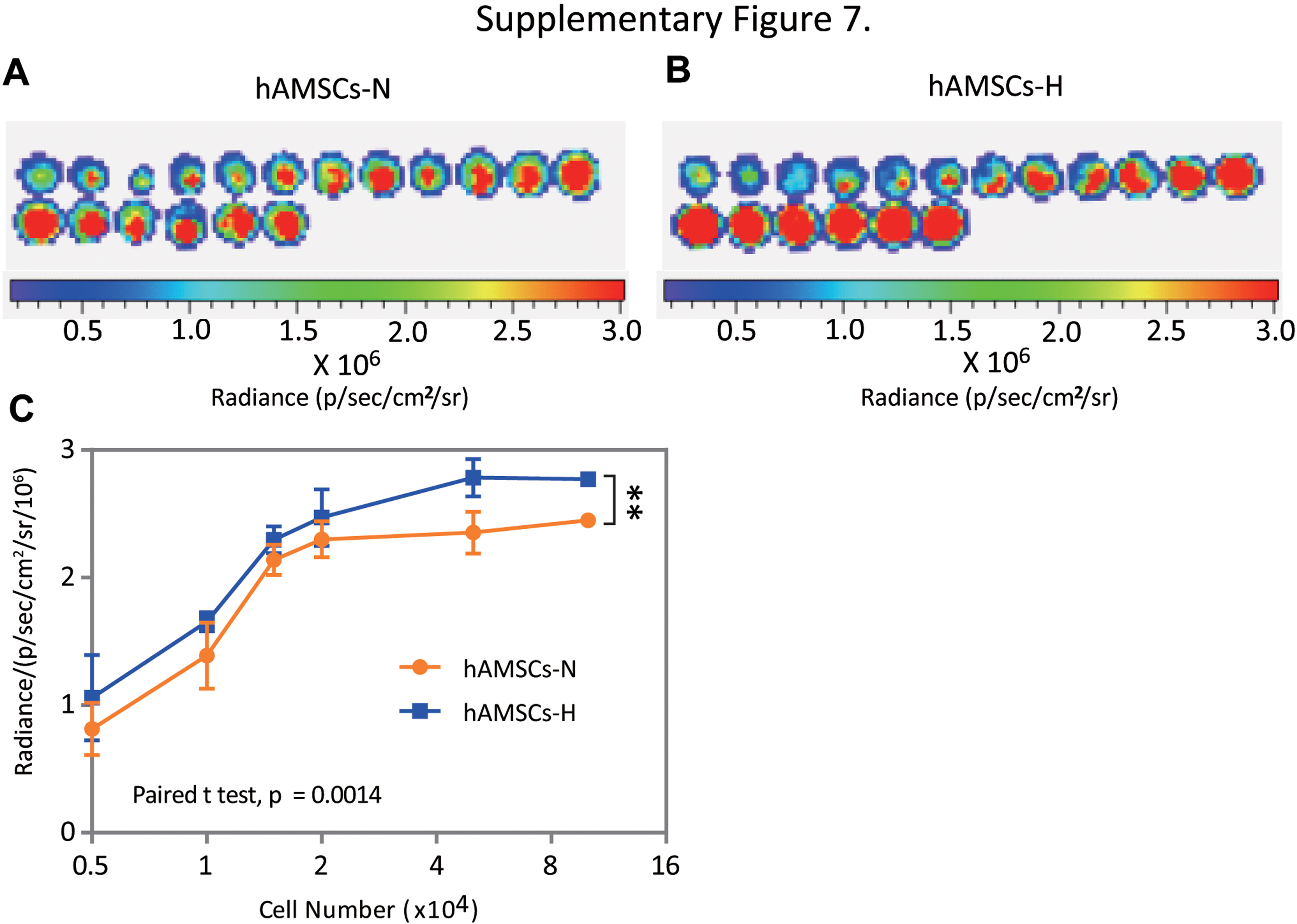

Supplement: Supplementary Figure 7 [file cddis2014521x7.tif]

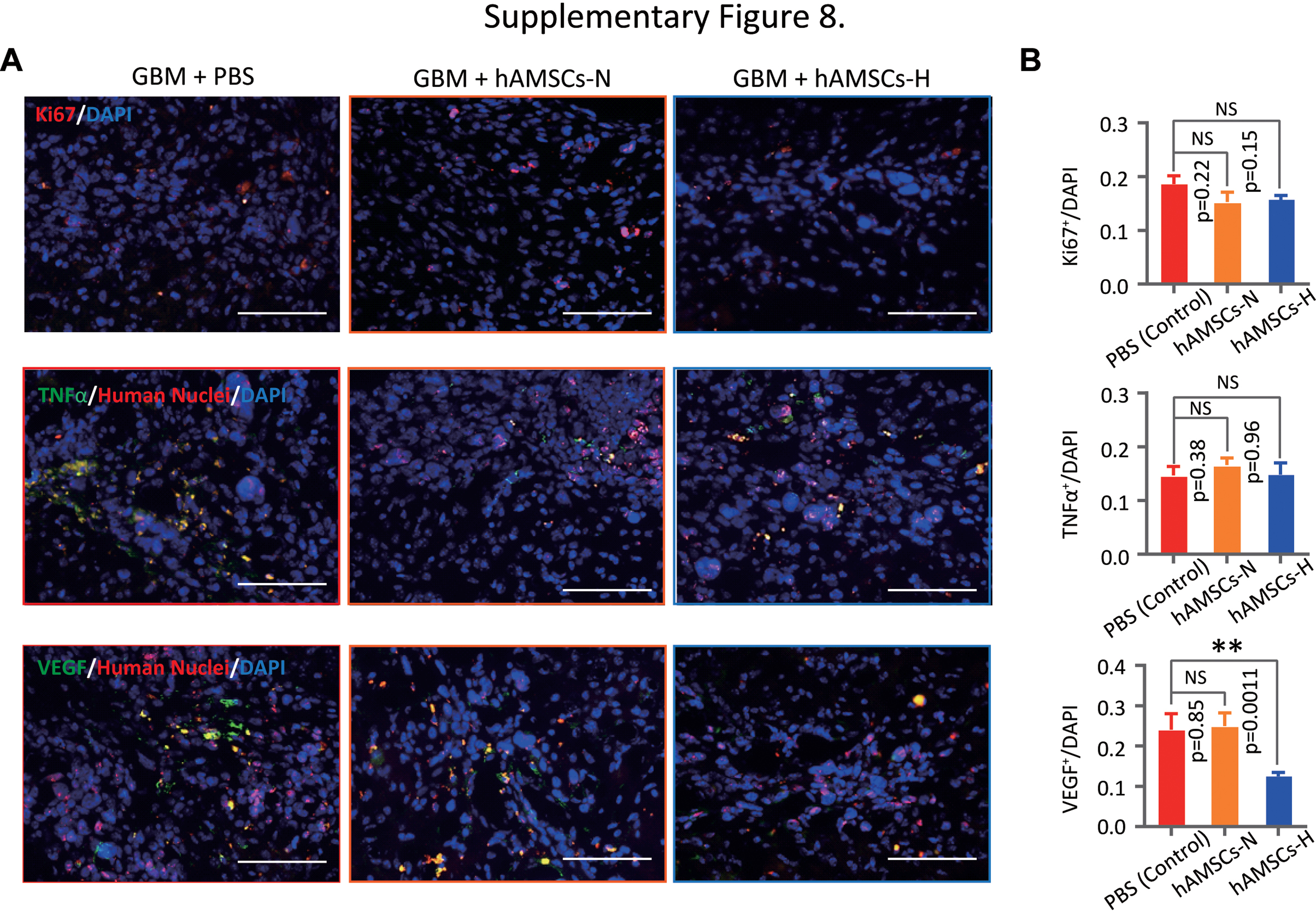

Supplement: Supplementary Figure 8 [file cddis2014521x8.tif]

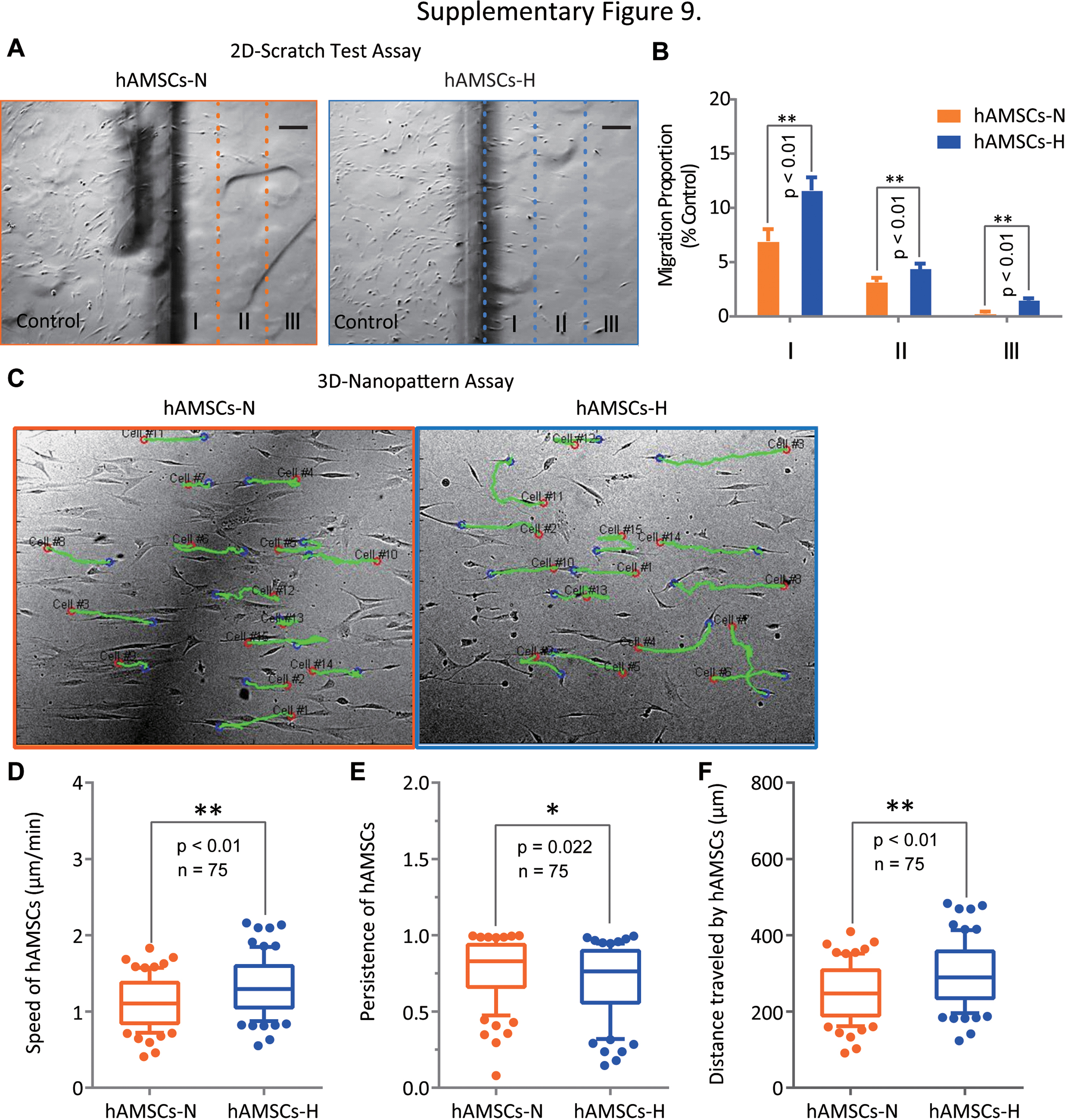

Supplement: Supplementary Figure 9 [file cddis2014521x9.tif]

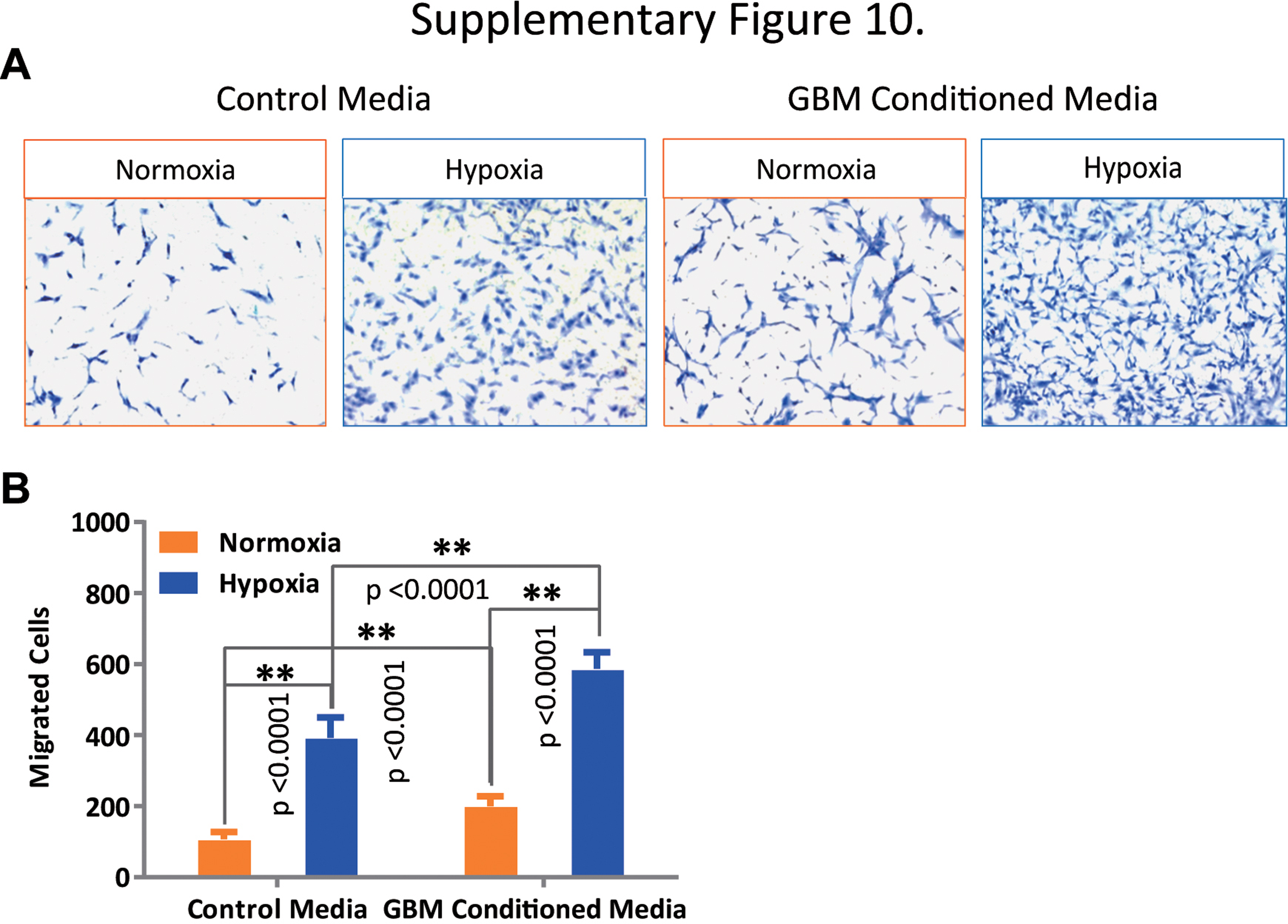

Supplement: Supplementary Figure 10 [file cddis2014521x10.tif]
