# Supplementary material for: Hypoxia-cultured human adipose-derived mesenchymal stem cells are non-oncogenic and have enhanced viability, motility, and tropism to brain cancer
Source: Cell Death Dis. 2014 Dec 11;5(12):e1567–. doi: 10.1038/cddis.2014.521 (PMC4649837; doi:10.1038/cddis.2014.521)
Supplement: Supplementary Information [file cddis2014521x11.doc]

**Supplementary Video Legends**

**Supplementary Video 1. Nanopattern migration assays of primary human adipose-derived mesenchymal stem cells (hAMSCs) cultured in normoxia and hypoxia. Hypoxia enhances migration ability of primary hAMSCs.** Time-lapse microscopy at magnification 4x showing movement of numerous primary hAMSCs cultured on a nanoridge-patterned, poly(urethane acrylate) substrate in complete, serum containing medium. Video depicts 6 hours.

**Supplementary Video 2. Nanopattern migration assays of commercial human adipose-derived mesenchymal stem cells (hAMSCs) cultured in normoxia and hypoxia. Hypoxia enhances migration ability of commercial hAMSCs.** Time-lapse microscopy at magnification 4x showing movement of numerous commercial hAMSCs cultured on a nanoridge-patterned, poly(urethane acrylate) substrate in complete, serum containing medium. Video depicts 6 hours.
